# Time course of serum cytokine level changes within 72 h after onset in children with acute encephalopathy and febrile seizures

**DOI:** 10.1186/s12883-022-03048-8

**Published:** 2023-01-07

**Authors:** Kazumi Tomioka, Masahiro Nishiyama, Shoichi Tokumoto, Hiroshi Yamaguchi, Kazunori Aoki, Yusuke Seino, Daisaku Toyoshima, Hiroshi Kurosawa, Hiroko Tada, Hiroshi Sakuma, Kandai Nozu, Azusa Maruyama, Ryojiro Tanaka, Kazumoto Iijima, Hiroaki Nagase

**Affiliations:** 1 Department of Pediatrics, Kobe University Graduate School of Medicine, Kobe, Hyogo Japan; 2 Department of Pediatric Critical Care Medicine, Hyogo Prefectural Kobe Children’s Hospital, Kobe, Hyogo Japan; 3 Department of Neurology, Hyogo Prefectural Kobe Children’s Hospital, Kobe, Hyogo Japan; 4 Department of Brain Development and Neural Regeneration, Tokyo Metropolitan Institute of Medical Science, Setagaya, Tokyo, Japan; 5 Department of Emergency and General Pediatrics, Hyogo Prefectural Kobe Children’s Hospital, Kobe, Hyogo Japan

**Keywords:** Cytokine, Acute encephalopathy, Febrile seizure, Children

## Abstract

**Background:**

Cytokine levels have been measured in acute encephalopathy (AE) to determine its pathology or as a diagnostic biomarker to distinguish it from febrile seizures (FS); however, the dynamics of cytokine level changes have not yet been fully captured in these two neurological manifestations. Thus, we aimed to explore the time course of serum cytokine level changes within 72 h after onset in AE and FS.

**Methods:**

We retrospectively measured cytokine level in residual serum samples at multiple timepoints in seven children whose final diagnoses were AE or FS.

**Results:**

The levels of 13 cytokines appeared to increase immediately after onset and peaked within 12–24 h after onset: interleukin (IL)-1β, IL-4 IL-5, IL-6, IL-8, IL-10, IL-17, eotaxin, fibroblast growth factor, granulocyte colony-stimulating factor, interferon gamma, interferon-inducible protein-10, and macrophage chemoattractant protein-1. There were no dynamic changes in the levels of three cytokines (IL-1 receptor agonist, macrophage inflammatory protein-1α, and platelet-derived growth factor-bb) 72 h after onset. Levels of some cytokines decreased to around control levels within 48 h after onset: IL-1β, IL-4, IL-5, IL-17, fibroblast growth factor, and interferon gamma. The levels of most cytokines appeared to be higher in AE, especially in hemorrhagic shock encephalopathy syndrome, than in FS.

**Conclusions:**

Cytokine levels in both AE and FS change dynamically, such as the levels of several cytokines increased within a few hours after onset and decreased at 12–24 h after onset. Therefore, it will be desirable to make clinical decisions regarding the administration of anti-inflammatory therapy in 24 h after onset in AE.

## Background

In most cases, children with seizures and/or impaired consciousness accompanied by fever with unknown etiology (SICF) are diagnosed with febrile seizures (FS) or acute encephalopathy (AE) [1]. Both of FS and AE are mostly associated with common infectious diseases such as influenza and exanthem subitem; there is a syndrome classification in AE such as acute necrotizing encephalopathy, acute encephalopathy with early biphasic seizures and later reduced diffusion (AESD), hemorrhagic shock and encephalopathy syndrome (HSES), and Reye-like syndrome [2, 3]. FS is a transient condition in which children do not experience sequelae, whereas AE is defined as impaired consciousness lasting longer than 24 h and is often associated with neurological sequelae [2]. Previous studies have indicated that the mortality rate among patients with AE is 6%, and many survivors exhibit motor and intellectual disabilities or epilepsy [2, 4]. Although FS and AE differ greatly in severity and outcomes, it is often difficult to distinguish AE from FS during the early stages of these diseases [2]. It has also been reported that some cases of AE progresses into a fatal condition within a few hours of onset [5], and also that early interventions may be effective in treating patients with AE [6, 7]. Therefore, it is necessary to identify a biomarker for distinguishing AE from FS within a few hours of onset.

The release of inflammatory cytokines is one of the major mechanisms involved in AE, hence, the anti-inflammatory treatment has been suggested [2]. There have been some previous reports on the use of cytokines as biomarkers for distinguishing AE from FS; however, their results were inconsistent [8–11]. The drawback with previous reports on cytokines in SICF was that the cytokine dynamics might not have been fully captured. A recent study involving an animal model for febrile status epilepticus reported an increase in hippocampal interleukin (IL)-1β levels 1–3 h after febrile status epilepticus onset with rapid decline over 24 h and return to baseline by 96 h; IL-6 levels peaked later at 24 h [12]. Consequently, the timing of sample acquisition is important in the case of febrile status epilepticus, as cytokine levels change over time [13]. However, data on cytokines reported in previous studies on AE and FS were limited owing to daytime sampling units (only one sample per day) [9, 11, 14–16].

Therefore, the purpose of this study was to explore the time course of serum cytokine level changes within 72 h after onset in SICF. Our hypothesis is that the levels of various serum cytokines would peak within 24 h after onset and that the levels of some cytokines would remain high after 24 h after onset.

## Methods

### Study design and subjects

This was a retrospective observational clinical study. It included seven children who were admitted to the pediatric intensive care unit at Kobe Children’s Hospital, Japan, between 2016 and 2017 because of SICF, and diagnosed finally as AE or FS, with residual samples stored more than once within 72 h after onset. We obtained clinical course-related information and imaging data of each patient from our clinical database and medical charts. Control samples were obtained from eight children who were diagnosed without epilepsy on examining the cause of developmental delay while they were physically healthy. All serum samples from the patients in the disease group were collected when clinicians determined that it was clinically necessary. Samples from patients in both disease and control groups were collected and frozen. We measured cytokine levels in 29 samples collected from the disease group and eight samples from the control group. The protocol and procedures of this retrospective observational study were approved by the Ethics Committees of Kobe University and Hyogo Prefectural Kobe Children’s Hospital. All experiments were performed in accordance with the relevant guidelines and regulations of these institutions and the Code of Ethics of the World Medical Association (Declaration of Helsinki). Informed consent was obtained from the patient’s parents in written form.

### Selection and measurement of cytokines

Cytokine profiling was performed using the Bio-Plex suspension array system and cytokine Human 27-Plex Panel (Bio-Rad Laboratories, Tokyo, Japan) according to the manufacturer’s instructions. We analyzed 16 cytokines: IL-1β, IL-1 receptor agonist (IL-1RA), IL-4, IL-5, IL-6, IL-8, IL-10, IL-17, eotaxin, fibroblast growth factor (FGF), granulocyte colony-stimulating factor (GCSF), interferon gamma (IFN-γ), interferon-inducible protein-10 (IP-10), macrophage chemoattractant protein-1 (MCP-1), macrophage inflammatory protein-1α (MIP-1α), and platelet-derived growth factor-bb (PDFG-bb). The following 11 cytokines with levels below the sensitivity of the tests conducted in patients of the disease group were excluded: IL-2; IL-7; IL-9; IL-12; IL-13; IL-15; granulocyte/macrophage colony-stimulating factor; vascular endothelial growth factor; MIP-1β regulated upon activation, normal T-cell expressed and secreted; and tumor necrosis factor alpha. The levels in all samples were measured in duplicate to improve accuracy. For statistical purposes, cytokine levels (pg/mL) below the lower limit of quantification were reported as the lower limit of quantification per analyte. In addition, cytokine levels above the upper limit of quantification were reported as the upper limit of quantification per analyte.

### Evaluation and statistical analysis

We evaluated the time course of the changes in the levels of 16 cytokines 72 h after onset according to the syndromes. We also compared the maximum levels of all types of cytokines at 0–23 h (less than 24 h) and 24–47 h after onset using the Mann–Whitney U test in six patients, with cytokines measured during both these time periods. Moreover, we investigated the relationship between cytokine levels and interventions targeted at IL-6. All statistical analyses were performed using EZR (Saitama Medical Center, Jichi Medical University, Saitama, Japan), which is a graphical user interface for R (The R Foundation for Statistical Computing, Vienna, Austria) [17].

### Clinical definitions and management

According to the criteria determined in our previous studies, we defined onset time as the time at which the initial manifestation of neurological symptoms, including convulsions or impaired consciousness, was first recognized by family members or other persons [1, 6, 18]. We defined FS as a seizure accompanied by fever (temperature ≥ 100.4 °F or 38 °C by any method), without central nervous system infection, that occurs in infants and children from 6 months to 14 years of age [19, 20]. We also defined AE as an impairment in consciousness of acute onset, with severity of Japan Coma Scale 20 or Glasgow Coma Scale < 11, and with duration of 24 h or longer according to the Guidelines for the diagnosis and treatment of acute encephalopathy in childhood.

[2]. Neurological sequelae were defined as the worsening of the Pediatric Cerebral Performance Category (PCPC) score [21]. HSES and AESD were diagnosed in accordance with previous reports [22, 23].

These patients were examined using laboratory investigations such as microbiological cultures of blood and cerebrospinal fluid, rapid assays for influenza virus, respiratory syncytial virus, and rotavirus, and real-time polymerase chain reaction for human herpes virus type 6 and 7; computed tomography imaging; magnetic resonance imaging; and electroencephalography (EEG). Clinical examinations and therapies were performed when clinicians determined that they were clinically necessary. Targeted temperature management (TTM) was performed according to a previous report [6]. High-dose steroid (HDS) treatment consisted of 30 mg/kg/day of methylprednisolone (maximum 1000 mg/day) for three continuous days.

## Results

### Patient background

Patient background is presented in Table 1. The patient group comprised four boys and three girls aged 1–9 years. The final diagnoses were HSES (three patients), AESD (two patients), and FS (two patients). Three patients had a history of neurological conditions (intellectual disability and hydrocephalus), and their PCPC was 3 before onset. The duration of fever before the manifestation of neurological symptoms was 0–35 h. Influenza A was detected in two patients. The initial neurological symptoms were convulsions in six patients and impaired consciousness in one patient. The duration of FS was 40–950 min. In case 7, impaired consciousness continued for 6 hours and non-convulsive status epilepticus was recognized during EEG-monitoring at hospital arrival; therefore, the duration of febrile seizure was maximum 360 minutes. All patients received intensive care, such as TTM and HDS treatment. The first HDS was administered 5–17 h after onset in five patients. Monoclonal antibody therapy for specific cytokine was not administered to any patient. Hemofiltration was not performed, but extracorporeal membrane oxygenation (ECMO) was induced in one patient with HSES 14 h after onset. TTM was started within 5–17 h after onset in all patients. Antiepileptic drugs, including thiamylal, midazolam, phenobarbital, fosphenytoin, and levetiracetam, were administered to all patients. Abnormalities were detected on brain magnetic resonance imaging in four patients. Neurological sequelae were detected in four patients (two with AESD and two with HSES).

**Table 1:** Patient background

Case	1	2	3	4	5	6	7
Sex	Female	Female	Male	Male	Female	Male	Male
Age (years)	3	5	9	1	3	1	8
Diagnosis	HSES	HSES	HSES	AESD	AESD	FS	FS
PCPC score before onset	3	1	1	3	1	3	1
Neurological history	Intellectual disability	None	None	Intellectual disability	None	Intellectual disability Hydrocephalus	None
Duration of fever before neurological symptom onset	0 h	1 day	1 day	1 day	0 h	1 h	1 day
Causative virus	Not detected	Influenza A	Not detected	Not detected	Not detected	Not detected	Influenza A
Initial neurological symptom	Convulsions	Convulsions	Convulsions	Convulsions	Convulsions	Convulsions	Impaired consciousness
Duration of seizures at onset (min)	950	211	40	333	99	181	Max. 360 (NCSE)
Treatment							
High-dose steroid treatment, start time	+ 17 h	+ 7 h	+ 15 h	–	–	+ 5 h	–
Targeted temperature management, start time	+ 17 h	+ 6 h	+ 15 h	+ 6 h	+ 12 h	+ 5 h	+ 12 h
Circulatory assist, start time	None	ECMO 14 h	None	None	None	None	None
Antiepileptic drugs	TA, MDL	MDL, PB	MDL, LEV	TA, LEV, PB	TA	TA	MDL, fPHT, TA
Abnormality on brain MRI	+ Atrophy	–	+ Bleeding Infarction	+ DWI high intensity in right hemisphere	+ T2 high intensity in right hemisphere	–	–
PCPC score 6 months after onset	4	1	5	4	2	3	1
Neurological sequelae	+	–	+	+	+	–	–

Abbreviations: *HSES* hemorrhagic shock encephalopathy syndrome, *AESD* acute encephalopathy with biphasic seizures and late reduced diffusion, *FS* febrile seizure, *PCPC* Pediatric Cerebral Performance Category, *NCSE* non-convulsive status epilepticus, *ECMO* extracorporeal membrane oxygenation, *TA* thiamylal, *MDL* midazolam, *PB* phenobarbital, *LEV* levetiracetam, *fPHT* fosphenytoin, *DWI* diffusion-weighted imaging, *MRI* magnetic resonance imaging

### Time course of serum cytokine level changes within 72 h after onset

**Fig. 1:**
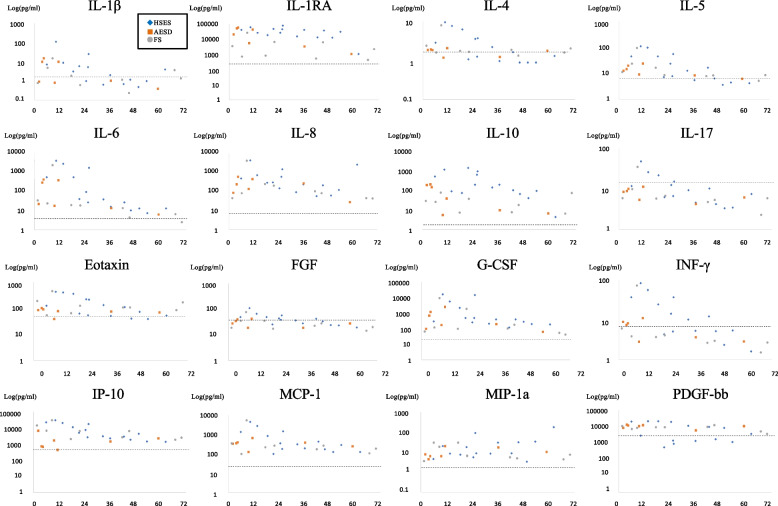
Cytokine levels at 72 h after onset in seven patients based on syndrome. The dotted line in the figure represents the reference value obtained from the control subjects with developmental delay (IL-1β, 1.4; IL-1RA, 228; IL-4, 1.4; IL-5, 6.2; IL-6, 3.3; IL-8, 6.0; IL-10, 1.6; IL-17, 13.5; eotaxin, 42.5; FGF, 34.2; GCSF, 20.0; IFN-γ, 6.8; IP-10, 427; MCP-1, 22.3; MIP-1α 1.6; and PDGF-bb, 2577). The cytokine levels in hemorrhagic shock and encephalopathy syndrome are marked as blue diamonds, acute encephalopathy with biphasic seizure and late reduced diffusion are marked as orange squares, and those in febrile seizure are marked as gray circles. The control data are expressed as average. Abbreviations: IL, interleukin; RA, receptor agonist; FGF, fibroblast growth factor; GCSF, granulocyte colony-stimulating factor; IFN-γ, interferon gamma; IP-10, interferon-inducible protein-10; MCP-1, monocyte chemoattractant protein-1; MIP-1α, macrophage inflammatory protein-1α; PDFG-bb, platelet-derived growth factor-bb

The time course of the changes in the levels of 16 cytokines is illustrated in Fig. 1. The levels of 13 cytokines appeared to increase immediately after onset and peaked within 12–24 h after onset: IL-1β, IL-4 IL-5, IL-6, IL-8, IL-10, IL-17, eotaxin, FGF, GCSF, IFN-γ, IP-10, and MCP-1. Among these, the levels of six cytokines—IL-1β, IL-4, IL-5, IL-17, FGF, and IFN-γ—decreased to around the control levels within 48 h after onset and those of the others remained continuously higher than the control levels until 72 h after onset. The levels of the remaining three cytokines—IL-1RA, MIP-1α, and PDFG-bb—were elevated beyond the control levels and did not tend to decrease until 72 h after onset.

Regarding the cytokine levels in relation to syndrome classification, although there were some cases involving exceptions, the levels of the aforementioned 13 cytokines, which appeared to peak within 24 h after onset, generally tended to be higher in HSES than in AESD and FS. IL-1RA levels increased not only in HSES but also in AESD and FS 24 h after onset. Both IL-1RA and IL-10 levels were higher in HSES than in AESD and FS 24 h after onset. We also compared the maximum levels of all types of cytokines at 0–23 and 24–47 h after onset (Table 2). The maximum level of IL-6 was significantly higher at 0–23 h than at 24–47 h after onset; therefore, it was confirmed that the IL-6 level peaked within 24 h after onset. Other differences were not statistically significant, but the median maximum levels of all the cytokines tended to be higher at 24–47 h than at 0–23 h after onset.

**Table 2:** Comparison of maximum cytokine levels within 0–23 h and 24–47 h after onset

Cytokine	Max cytokine level in 0–23 h		Max cytokine level in 24–47 h		*P* value
	Median	Range	Median	Range	
IL-1β (pg/mL)	11.4	(4.6–107.8)	2.3	(1.0–24.5)	0.07
IL-1RA (pg/mL)	37,173.2	(764.0–60,163.6)	9261.1	(481.4–78,583.5)	0.39
IL-4 (pg/mL)	4.7	(1.1–9.2)	1.9	(1.2–3.6)	0.39
IL-5 (pg/mL)	56.1	(7.1–106.8)	8.0	(7.6–53.5)	0.23
IL-6 (pg/mL)	1165.8	(21.3–3438.7)	18.4	(4.0–1491.2)	0.04
IL-8 (pg/mL)	612.2	(227.5–4176.7)	193.2	(75.8–1408.7)	0.13
IL-10 (pg/mL)	126.8	(23.0–1196.8)	93.4	(7.0–807.0)	0.31
IL-17 (pg/mL)	22.9	(6.4–405.2)	6.2	(4.2–222.5)	0.18
Eotaxin (pg/mL)	111.4	(62.1–438.0)	105.2	(53.5–222.5)	0.82
FGF (pg/mL)	52.5	(24.6–108.6)	32.5	(17.9–55.5)	0.18
GCFS (pg/mL)	4104.2	(114.1–16,389.2)	223.7	(101.2–14,969.2)	0.20
IFN-γ (pg/mL)	34.0	(3.8–84.4)	4.4	(2.6–37.1)	0.13
IP-10 (pg/mL)	12,996.3	(663.3–29,227.7)	4252.8	(1346.0–17,348.6)	0.31
MCP-1 (pg/mL)	1701.6	(96.9–5238.8)	303.3	(159.6–1444.0)	0.31
MIP-1α (pg/mL)	17.9	(8.1–28.3)	8.4	(4.6–87.6)	0.18
PDGF-bb (pg/mL)	11,168.3	(551.1–21,539.5)	7328.7	(893.3–19,659.5)	0.39

Data are expressed as median (range)

Abbreviations: *IL* interleukin, *RA* receptor agonist, *FGF* fibroblast growth factor, *GCSF* granulocyte colony-stimulating factor, *IFN-γ* interferon gamma, *IP-10* interferon-inducible protein-10, *MCP-1* monocyte chemoattractant protein-1, *MIP-1*α macrophage inflammatory protein-1α, *PDFG-bb* platelet-derived growth factor-bb

### Relationship between cytokines and interventions targeted at IL-6

**Fig. 2:**
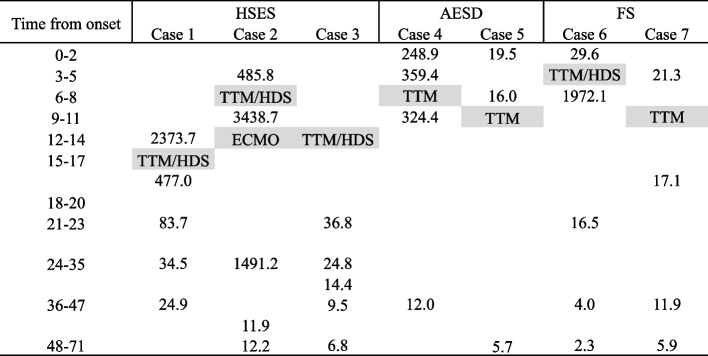
Time series of IL-6 level changes and interventions at 72 h after onset. The cytokine levels are measured in pg/mL. Abbreviations: IL, interleukin; TTM, targeted temperature management; HDS, high-dose steroid; ECMO, extracorporeal membrane oxygenation

The findings related to this relationship are illustrated in Fig. 2. TTM and/or HDS were administered within 12 h after onset in five patients (cases 2, 4, 5, 6, and 7). IL-6 levels decreased 3 h after TTM and/or HDS administration in cases 1 and 4. In contrast, IL-6 levels increased 3 h after TTM and HDS administration in cases 2 and 6. Changes within a few hours after TTM and/or HDS administration were not constant, but the cytokine levels decreased 24 h after TTM/HDS administration even in cases involving neurological sequelae. The cytokine levels also decreased without HDS administration 24 h after onset in case 4.

## Discussion

This study showed that cytokine levels changed dynamically during the early stage of SICF, including AE and FS, within 72 h after onset. The levels of most cytokines were elevated immediately after onset, and those of several cytokines (IL-1β, IL-4, IL-6, IL-8, IL-17, eotaxin, FGF, IFN-γ, and MCP-1), including pro-inflammatory cytokines, peaked within 12 h after onset and decreased to around the control levels within 48 h after onset. Conversely, the levels of some cytokines, such as IL-1RA and IL-10, which are considered anti-inflammatory cytokines, did not decrease within 24 h after onset. This was not different from the findings of a previous report indicating that IL-10 and IL-1RA levels increased in FS [24].

In the present study, we made the final diagnosis according to the diagnostic criteria of Guidelines for the diagnosis and treatment of acute encephalopathy in childhood [2], and found that cytokine levels were elevated not only in AE but also in FS. A previous study reported that cytokine levels were elevated in FS [25, 26]. Regarding AE, inflammatory cytokines are thought to play an important role in severe AEs, such as HSES and acute necrotizing encephalopathy [2]. In contrast, the main mechanisms underlying AESD have been considered excitotoxic [2]. Reports have revealed that the levels of several cytokines are not elevated or are slightly elevated in AESD [8, 14, 15]. In the present study, the levels of some cytokines were elevated in AESD and FS and were as high as those in HSES. This result differs from that of previous reports. The reason for the elevated cytokine levels in AESD or FS might be because they were measured during the earlier stages than those in previous studies. Alternatively, pathogenic differences are a possible cause of elevated cytokine levels in AESD or FS because AE in children is generally thought to develop during the course of infectious diseases. In addition, cytokine levels were reported to be higher in HSES than in AESD [14, 15]; however, it was suggested that cytokine levels change dynamically over time, so they should be measured and compared regularly when used for diagnosis. The results of this study did not allow us to distinguish between AE and FS suggesting that AE and FS are overlapping syndromes representing a neuroinflammatory disorders triggered by fever mainly associated infections. Pensato et al. claimed that many febrile infection-associated encephalopathies including AESD, HSES and other syndromes showing overlapping clinical-radiological features and, presumably, similar pathogenesis. They said that those febrile infection-associated encephalopathies shared the same pathogenic mechanism of cytokine-mediated neuroinflammatory process, but to date, those above encephalopathies have been defined based on clinical presentation associated with specific investigative findings, irrespective of the pathogenic mechanisms [27]. Although FS was not mentioned in the study, it is possible that FS is also included in the spectrum of these encephalopathy disorders, as FS is triggered by febrile infection.

In clinical practice, the detailed time course of cytokine level changes in AE will aid in making clinical decisions regarding the administration of anti-inflammatory therapy. Based on our observations, it might be better to start anti-inflammatory treatment, such as steroid administration, within 12 h after onset because pro-inflammatory cytokine levels increase within 12–24 h after onset. The present study could not investigate whether HDS, TTM, or ECMO were effective in treating SICF because the disease severity and received therapies were different in each patient. Cytokine levels did not necessarily decrease within a few hours after the intervention with HDS or TTM. Moreover, some cytokine levels were high even after 12 h after onset; therefore, we suppose that anti-inflammatory treatments need to be continued after 12 h after onset. Though monoclonal antibody therapy for specific cytokine was not administered in patients in this study, it may be useful for patients whose cytokine levels are elevated. In future studies, we plan to compare cases with and without the administration of interventions.

This study has some limitations. Specimens were obtained by random blood sampling, and the sampling time was predetermined. The sample size was too small to show the exact dynamics of cytokines in different time point. Therefore, there is a need to perform the investigation in a larger number of patients. Because of the bias associated with anti-inflammatory treatment, the dynamics of natural cytokine levels are unknown and may have been modified. We also did not compare cytokine levels of AE and FS in this study, so the comparative study with a large number of cases is necessary in the future.

## Conclusion

Cytokine levels in both AE and FS change dynamically from minutes to hours, such as the levels of several cytokines upstream of inflammation increased within a few hours after onset and decreased at 12–24 h after onset. Detailed knowledge of the time course of cytokine level changes will aid in making clinical decisions regarding the administration of anti-inflammatory therapy in 24 h after onset in AE. Although our study could not differentiate AE and FS by cytokine levels, but the results suggested that AE and FS may be neuroinflammatory spectrum disorders associated with fever because cytokine levels increased in all syndromes.

## Data Availability

The data sets in this study are available from the corresponding author upon reasonable request.

## Abbreviations

AE: acute encephalopathy
AESD: acute encephalopathy with biphasic seizures and late reduced diffusion
ECMO: extracorporeal membrane oxygenation
FS: febrile seizures
FGF: fibroblast growth factor
GCSF: granulocyte colony-stimulating factor
HSES: hemorrhagic shock encephalopathy syndrome
HDS: High-dose steroid
IFN-γ: interferon gamma
IP-10: interferon-inducible protein-10
MCP-1: macrophage chemoattractant protein-1
MIP-1α: macrophage inflammatory protein-1α
PCPC: Pediatric Cerebral Performance Category
PDFG-bb: platelet-derived growth factor-bb
SICF: seizures and/or impaired consciousness accompanied by fever
TTM: targeted temperature management.
